# Draft Genome Sequence of a Biodesulfurizing Bacterium, *Gordonia* sp. Strain IITR100

**DOI:** 10.1128/genomeA.00230-17

**Published:** 2017-04-27

**Authors:** Jananee Jaishankar, Pooja Singh, Preeti Srivastava

**Affiliations:** Department of Biochemical Engineering & Biotechnology, Indian Institute of Technology Delhi, New Delhi, India

## Abstract

We report here the whole-genome sequence of a biodesulfurizing bacterium, *Gordonia* sp. strain IITR100. The bacterium has the unique ability to desulfurize both aliphatic and aromatic organosulfurs. The draft genome sequence will provide insights into the various genes and regulators involved in biodesulfurization and other catabolic pathways.

## GENOME ANNOUNCEMENT

*Gordonia* sp. strain IITR100 was isolated from petroleum-contaminated soil by enrichment culture using 4,6-dimethyl dibenzothiophene as the sole sulfur source (1). The bacterium could desulfurize 76% sulfur from crude oil and resulted in about a 31% decrease in the viscosity of heavy crude oil (2). The whole-genome sequence was essential to understand not only the different genes involved in biodesulfurization and their mechanism of regulation but also to explore other catabolic abilities of the bacterium.

To determine the whole-genome sequence, genomic DNA was isolated, and a paired-end library was constructed using Illumina TruSeq Nano DNA LT library preparation kit. The mean library fragment size was 593 bp. The library was sequenced using Illumina NextSeq 500 using 2 × 150 chemistry. Trimmomatic version 0.35 was used to remove the adapter sequences, ambiguous reads (reads with unknown nucleotides “N” larger than 5%), and low-quality sequences (reads with more than 10% quality threshold [QV] <20 Phred score) (3). The total number of reads obtained was 5,335,899. The filtered high-quality reads were assembled into 56 scaffolds using SPAdes (version 3.7.1) (4). The total size of assembly was obtained as 5,285,492 bp. The average size of scaffolds was 88,143 bp, with an *N*_50_ length of 223,109 bp. The scaffold with the smallest size was 527 bp, and the scaffold with the biggest size was 827,323 bp.

Genes were predicted from the assembled scaffolds using the Prodigal software, with default parameters (5). A total of 4,766 genes were predicted. The average gene length was 1,006 bp. The length of the smallest gene was 75 bp and the maximum gene length was 41,934 bp. Functional annotation of the genes was performed using Blastx program, which is a part of NCBI Blast 2.3.0+ standalone tool. Out of the 4,766 genes, 4,703 genes found hits, while 63 genes were not annotated against the nonredundant (NR) protein database. The Blast results revealed the closest homology to *Gordonia* sp. strain SGD-V-85.

Gene ontology (GO) annotations of the predicted genes were determined by the Blast2GO program. GO assignments were used to classify the functions of the predicted genes. GO mapping also provided ontology of gene product properties, which are grouped into three main domains: biological process, molecular function, and cellular component. The GO distribution of the predicted genes was found to be highest for molecular function (2,144 genes), followed by biological process (1,927 genes) and cellular component (1,050 genes).

The gene annotation analysis of *Gordonia* sp. strain IITR100 revealed the presence of five genes involved in the biodesulfurization process, which includes 2-hydroxybiphenyl-2-sulfinate desulfinase, dibenzothiophene (DBT) desulfurization enzyme, DBT monooxygenase, sulfite reductase, and flavin reductase. Interestingly, genes for resistance to antibiotics (five genes) and metal (14 genes), including arsenic, tellurium, chromate, copper, etc., were also detected. The genes playing a regulatory role, including monooxygenases (44 genes), starvation protein (three genes), and heat, cold, and alkaline shock proteins (12 genes), were also annotated in the genome of this organism.

### Accession number(s).

The draft genome sequence of *Gordonia* sp. strain IITR100 has been deposited at the EMBL/DDBJ/Genbank under accession number MVKV00000000. The version described in this paper is version MVKV01000000.
